# Surgical Management of Pseudophakic Malignant Glaucoma via Anterior Segment-Peripheral Iridectomy Capsulo-Hyaloidectomy and Anterior Vitrectomy

**DOI:** 10.1155/2012/794938

**Published:** 2012-10-14

**Authors:** Isıl Basgil Pasaoglu, Cigdem Altan, Sukru Bayraktar, Banu Satana, Berna Basarir

**Affiliations:** Beyoglu Eye Education and Research Hospital, Beyoglu, Istanbul, Turkey

## Abstract

*Purpose*. To describe our surgical technique in the management of pseudophakic malignant glaucoma refractory to conventional treatment. *Methods*. Two pseudophakic eyes with malignant glaucoma underwent peripheral iridectomy, lens capsulectomy, hyaloidectomy, and anterior vitrectomy through a clear corneal incision by using a vitreous cutter. *Results*. Prompt resolution of malignant glaucoma was achieved in both cases and no recurrence was observed during postoperative followup of five months. *Conclusions*. An anterior segment surgeon can treat pseudophakic malignant glaucoma successfully by using a vitreous cutter inserted through a corneal incision and performing peripheral iridectomy, capsulo-hyaloidectomy, and anterior vitrectomy.

## 1. Introduction

Malignant glaucoma or aqueous misdirection syndrome is described as elevated intraocular pressure (IOP) and a uniform flattening of the central and peripheral anterior chamber in the presence of a patent iridotomy. It occurs most often after filtration surgery in eyes with angle closure glaucoma [1–3] but has also been described after cataract extraction [4], laser iridotomy [5], capsulotomy [6], cyclophotocoagulation [7], and initiation of topical miotic therapy. 

Though relatively uncommon, its management has usually been challenging. Medical therapy with cycloplegics, aqueous suppressants, and hyperosmotic agents has been the standard initial treatment. In pseudophakic eyes refractory to the above medical treatment, neodymium : yttrium-aluminum-garnet (Nd : YAG) laser posterior capsulotomy and hyaloidotomy and pars plana vitrectomy (PPV) have been used with variable success [1, 2, 8]. 

In this case study, we aimed to present our surgical approach in the management of pseudophakic malignant glaucoma which consists of peripheral iridectomy, zonulectomy, hyaloidectomy, and anterior vitrectomy performed by an anterior segment surgeon using a vitreous cutter inserted through a clear corneal incision.

## 2. Surgical Procedure

**Figure 1:**
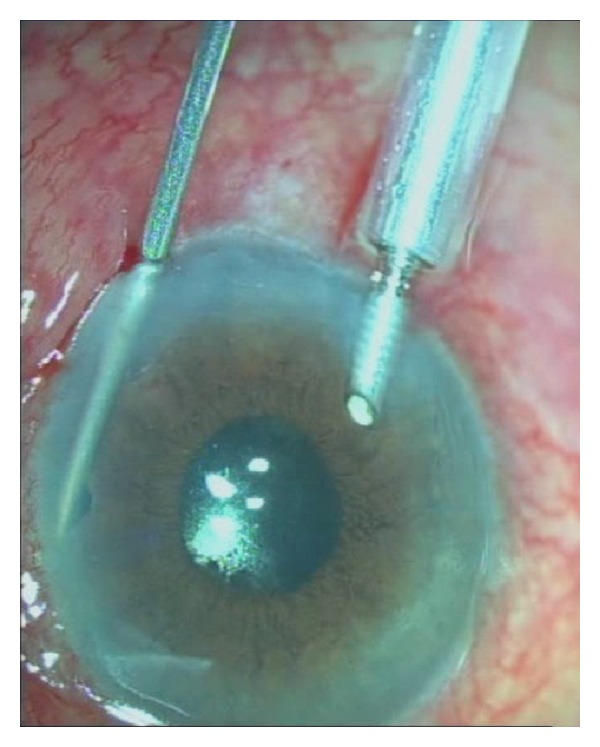
Peripheral iridectomy, capsulo-hyaloidectomy, and anterior vitrectomy procedure in malignant glaucoma.

Both surgeries were performed by the same anterior segment surgeon (SB). Sub-Tenon's anesthesia was used in both of the study eyes. An inferotemporal clear corneal incision was made with a 20-gauge MVR knife, and an anterior chamber maintainer cannula was inserted. It was connected to an infusion bottle full of balanced salt solution. The bottle height was adjusted in order to deepen the anterior chamber slightly but to avoid increasing IOP dangerously. A second clear corneal incision was made in the superotemporal quadrant by using the same knife. The incision was so constructed that the tip of the knife targeted the peripheral iris at 12 o'clock. A vitreous cutter was then inserted into the anterior chamber and an opening was made in the peripheral iris at the superior quadrant (Figure 1). The opening had to be sufficiently large (approximately 2 mm in diameter) and as peripheral as possible. Then the cutter was advanced into the already created opening and another cut was performed at the lens capsule under the iridectomy. Finally the anterior hyaloid face, and anterior vitreous were removed by using the cutter in order to eliminate the blockade and aqueous misdirection completely. The sufficient amount of vitreous excision was confirmed by the observation of sudden iris movement and deepening of the anterior chamber. The corneal incisions were closed with 10.0 nylon sutures.

**Case 1**

**Figure 2:**
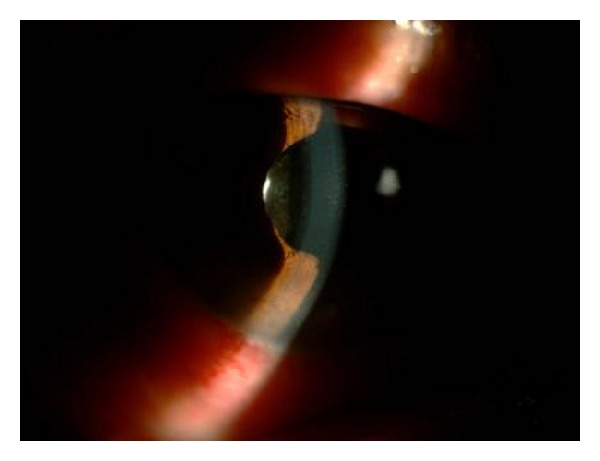
Preoperative slit-lamp photograph of the right eye in Case 1. A shallow anterior chamber was present.

**Figure 3:**
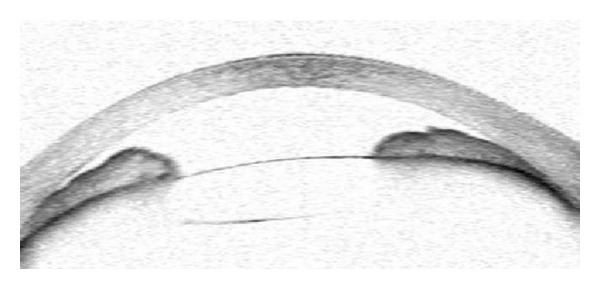
Preoperative anterior chamber optic coherence tomography picture of the right eye in Case 1. Anterior chamber depth was 2.10 mm.

A 70-year-old woman with a history of angle-closure glaucoma and cataract surgery presented with malignant glaucoma in her right eye approximately one month following trabeculectomy. She had lost the fellow eye because of glaucoma. She had a patent laser iridotomy and visual acuity was 2/10 (Snellen) in that eye. After the IOP rise to 35 mm Hg in the third postoperative week of trabeculectomy surgery, the IOP was achieved at 16 mm Hg with 3 glaucoma medications, a shallow central and peripheral anterior chamber was noted (Figure 2). Topical steroids and cycloplegics were prescribed. Anterior chamber depth was measured as 2.10 mm and axial length as 21.26 mm, with IOLMaster optical biometry (Carl Zeiss Meditec AG, Germany). Anterior chamber optic coherence tomography (Visante OCT 3.0 Model 1000, Carl Zeiss Meditec, Inc.) demonstrated convex iris configuration, closed angle, and shallow anterior chamber in the right eye (Figure 3). Fundus examination and B-scan ultrasonography ruled out the presence of suprachoroidal hemorrhage.

**Figure 4:**
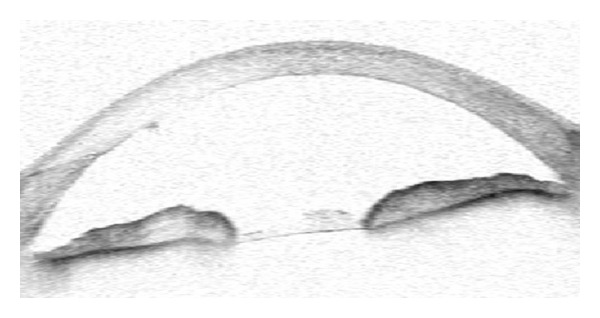
Postoperative anterior chamber optic coherence tomography picture of the right eye in Case 1. Anterior chamber depth was 2.80 mm.

**Figure 5:**
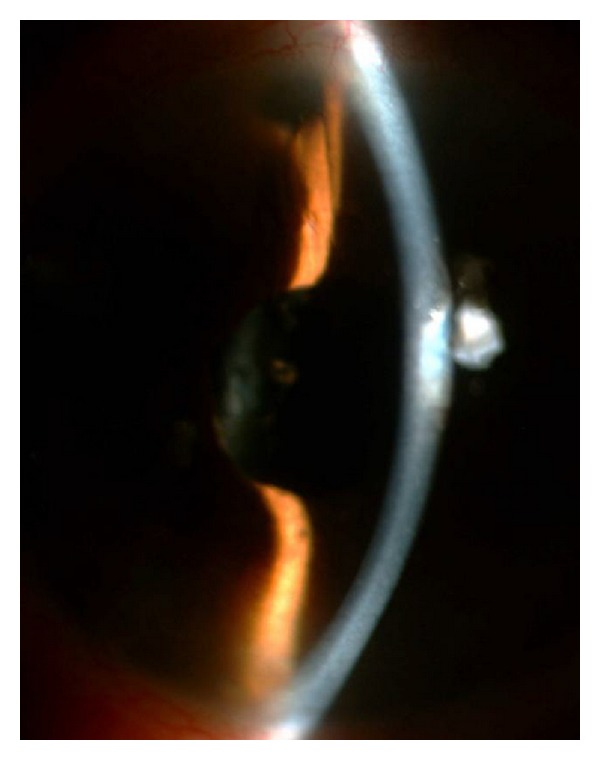
Postoperative slit-lamp photograph of the right eye in case 1. Anterior chamber was deep in the presence of a patent iridectomy, capsulo-hyaloidectomy.

A peripheral iridectomy, capsulectomy, hyaloidectomy, and anterior vitrectomy were performed by using a vitreous cutter as described above. The IOP was measured as 10 mm Hg on the first postoperative day. One week after surgery, IOP was 5 mm Hg and the anterior chamber remained deep (Figure 4). One month after the surgery, visual acuity improved to 5/10 (Snellen), the anterior chamber was deep, and the IOP was 8 mm Hg without glaucoma medication. There was no IOP rise or shallowing chamber in the last five months' controls (Figure 5).

**Case 2**

A 70-year-old male was presented with shallow anterior chamber and IOP above 30 mm Hg in his left eye. He had no previous history of glaucoma and reported to have uncomplicated phacoemulsification and intraocular lens implantation in his left eye in another clinic. The patient noticed pain and reduction of visual acuity one week postoperatively, the visual acuity was perception of hand motions. Cornea was edematous and there was not any choroidal detachment and/or suprachoroidal hemorrhage.

Malignant glaucoma was diagnosed and a 23-gauge pars plana vitrectomy (PPV) combined with posterior capsulotomy and peripheral iridectomy was performed as the IOP could not be controlled with antiglaucoma drugs, topical cycloplegics and prednisolone acetate. The anterior chamber deepened and IOP was 18 mm Hg on the first postoperative day. Ten days later, IOP was 8 mm Hg with topical dorzolamide-timolol fixed combination twice daily. The corneal edema was persisted.

Nine months after PPV, recurrence of malignant glaucoma was noted with an IOP of 34 mm Hg with significant central anterior chamber shallowing. Peripheral iridectomy, capsulectomy, hyaloidectomy and anterior vitrectomy by using a vitreous cutter inserted through a clear corneal incision were performed. The sudden deepening of anterior chamber was observed during surgery and the IOP was measured as 10 mm Hg on the first postoperative day. One week after surgery, the anterior chamber was deep and IOP was 14 mm Hg. At the postoperative 5th-month visit, IOP was 16 mm Hg without medications, anterior chamber was deep, and the peripheral iridectomy and capsulo-hyaloidectomy were patent.

## 3. Discussion

Malignant glaucoma, first described by Von Graefe in 1869, is characterized by elevated IOP with a shallow or flat anterior chamber [9]. Although the exact mechanism of malignant glaucoma remains unclear, an alteration in the anatomic relationship of the lens, ciliary body, and anterior hyaloid face causing an aqueous misdirection and blockade was suggested in the pathogenesis. The aqueous has been thought to be entrapped inside the vitreous cavity as aqueous pockets resulting in forward movement of the iris-lens diaphragm which causes the secondary angle closure glaucoma.

Malignant glaucoma occurs in 2 to 4 percent of eyes undergoing surgery for angle-closure glaucoma. It may occur within hours to days or years after trabeculectomy, cataract extraction with or without IOL implantation, glaucoma drainage implantation, laser iridotomy, capsulotomy, laser suture lysis or argon laser photocoagulation, miotic therapy, needling of filtering blebs, viscoelastic use, or intravitreal injection [10]. In our first patient malignant glaucoma developed following trabeculectomy while cataract surgery preceded the onset of aqueous misdirection in the second case.

Medical therapy with topical beta-blockers, alpha-adrenergic agonists, topical and oral carbonic anhydrase inhibitors, osmotic agents including oral glycerol or isosorbide or intravenous mannitol, and cycloplegic agents should be the first line of treatment in malignant glaucoma [11]. The goal is to decrease aqueous humor production, shrink the vitreous body, and move the iris-lens diaphragm backward. Approximately fifty percent of cases were reported to respond medical therapy, but recurrences were reported to be common following cessation of cycloplegics [12].

Nd : YAG laser capsulotomy/hyaloidotomy has been used in aphakic or pseudophakic malignant glaucomas with varying degrees of success. Its mechanism was proposed to relieve the blockade and reverse the aqueous misdirection [8, 13–16]. Also, direct argon laser application through a peripheral iridectomy has been used in an attempt to shrink the ciliary processes and relieve the cilio-lenticular block to anterior flow of aqueous [17, 18]. Herschler reported success in five of six eyes treated in this manner [18]. Transscleral diode laser photocoagulation also has been used in the treatment of malignant glaucoma by reducing aqueous production and its flow [19].

In those cases refractory to medical and/or laser treatment, surgical treatment has to be used. PPV has been reported to treat pseudophakic malignant glaucoma in 67% to 100% of cases [1, 2]; however, it requires vitreoretinal surgical expertise and cannot be performed easily and safely by an anterior segment surgeon. Vitrectomy has been thought to prevent aqueous accumulation inside the vitreous cavity [2]. However, this may not be enough to break the cycle of malignant glaucoma because the ciliolenticular aqueous blockade cannot be completely eliminated by removing the central vitreous only and aqueous accumulation may continue despite the procedure. Sharma et al. described the recurrence-free follow-up period ranging from 5 to 32 months with a vitrectomy-phacovitrectomy procedure [20]. In a recent retrospective series, the relapse rate was 100% after a medical therapy, 75% after a YAG laser capsulotomy and a hyaloidotomy, 75% after a conventional vitrectomy, and 66% after an anterior vitrectomy in combination with an iridectomy-zonulectomy [21]. In our study, the second case had a recurrence nine months following a 23-Gauge PPV-posterior capsulotomy. It was postulated that all of the tissues (iris, lens capsule, anterior hyaloid, and anterior vitreous) had to be removed completely in order to create a permanent passage between the anterior chamber and the vitreous cavity, a task which was not easy to be accomplished by PPV alone.

In our study, two cases of pseudophakic malignant glaucoma were treated successfully by using an anterior chamber approach consisting of a capsulo-hyaloidectomy and anterior vitrectomy performed through a peripheral iridectomy, creating a permanent passage between the anterior chamber and vitreous cavity by eliminating the aqueous misdirection. We observed sudden deepening of the anterior chamber and elimination of blockade in both cases during surgery. We think that it is not only necessary for the success of the surgery, but also helps us to understand the pathogenesis of the disease better. Debrouwere et al. emphasized that total vitrectomy was not effective in 66% of their patients unless an zonulectomy was added to the procedure [21]. The necessity of the establishment of a permanent passage between the anterior chamber and vitreous cavity was well demonstrated in another study, all of the five pseudophakic patients were successfully treated with a combined pars plana anterior vitrectomy, hyaloidectomy, zonulectomy, and peripheral iridectomy, and no recurrence was observed [22]. However, the vitreous cutter had to be inserted through a pars plana incision in their technique which was a rather blind and more dangerous technique than that of the safer anterior chamber approach used in our patients.

Malignant glaucoma was not recurred in our patients. The procedure may not only prevent recurrences but also may be helpful in the achievement of long-term IOP control. It has the advantage of a shorter operation time, and appears to be technically easier and potentially safer to be used for the anterior segment surgeon.

Malignant glaucoma is relatively a rare disease, which makes it difficult to collect a large group of patients. Despite the small number of patients and short duration of follow-up, we believe that zonulectomy, hyaloidectomy, and anterior vitrectomy procedure performed through a peripheral iridectomy by using a vitreous cutter via clear corneal incision was a valuable option in the management of pseudophakic malignant glaucoma.
